# Outcome-Specific Performance of HALP, AIMS65, Glasgow–Blatchford, and Rockall Scores in Emergency Department Patients with Acute Upper Gastrointestinal Bleeding: A Retrospective Cohort Study

**DOI:** 10.3390/jcm15208196

**Published:** 2026-10-21

**Authors:** Esat Barut, İbrahim Halil Yasak, Eyyup Sabri Şeyhanlı, Ramazan Giden, Kemal Barut

**Affiliations:** 1 Department of Emergency Medicine, Faculty of Medicine, Harran University, Sanliurfa 63300, Türkiye; 2 Department of Emergency Medicine, Mehmet Akif İnan Training and Research Hospital, Sanliurfa 63300, Türkiye; 3 Department of Emergency Medicine, Gazi Yaşargil Training and Research Hospital, Diyarbakir 21070, Türkiye

**Keywords:** upper gastrointestinal bleeding, HALP, AIMS65, Glasgow–Blatchford, Rockall, emergency department

## Abstract

**Background/Objectives**: The Hemoglobin–Albumin–Lymphocyte–Platelet (HALP) score integrates hematologic, nutritional, immune, and platelet parameters, but its prognostic value in acute upper gastrointestinal bleeding (UGIB) remains uncertain. We compared the performance of HALP with AIMS65, Glasgow–Blatchford Score (GBS), and Rockall scores. **Methods**: We retrospectively studied consecutive adults with endoscopy-confirmed UGIB presenting to the emergency department from January 2021 through December 2024. Outcomes were in-hospital mortality, packed red blood cell (PRBC) transfusion within 24 h, and composite severe UGIB, defined as ≥2 PRBC units transfused within 24 h or UGIB-related intensive care unit admission. Discrimination was assessed using receiver operating characteristic curves and areas under the curve (AUCs); pairwise comparisons used the correlated-sample DeLong test. Firth-penalized logistic regression assessed the adjusted association between HALP and in-hospital mortality. **Results**: Of 209 patients, 24 (11.5%) died and 36 (17.2%) had variceal bleeding. For mortality, AUCs were 0.580 for inverse HALP, 0.802 for AIMS65, 0.733 for GBS, and 0.731 for Rockall. For 24-h PRBC transfusion, AUCs were 0.629, 0.642, 0.814, and 0.511, respectively; for severe UGIB, they were 0.622, 0.594, 0.781, and 0.501. No statistically significant adjusted association between HALP and mortality was observed (aOR, 0.91 per 10-point increase; *p* = 0.571). **Conclusions**: HALP showed limited discrimination across the evaluated UGIB outcomes, and no statistically significant adjusted association with mortality was observed. AIMS65 had the highest observed AUC point estimate for mortality, although its AUC did not differ significantly from those of GBS or Rockall. GBS had the highest AUCs for PRBC transfusion and severe UGIB. These findings support outcome-specific selection of UGIB risk scores and do not support the use of HALP as a stand-alone risk-stratification tool without external validation.

## 1. Introduction

Upper gastrointestinal bleeding (UGIB) is a common emergency condition associated with substantial morbidity and mortality [1,2]. Risk factors associated with mortality include advanced age, low hemoglobin levels, hypotension, comorbidities, rebleeding, and hypoalbuminemia [2,3]. Therefore, early risk classification and rapid identification of high-risk patients are critical for determining appropriate treatment strategies.

For this purpose, various clinical scoring systems have been developed. The Rockall score was developed specifically to predict mortality, taking into account age, hemodynamic parameters, comorbidities, and endoscopic findings [4]. The Glasgow–Blatchford Score (GBS) is based on clinical and laboratory parameters and was initially developed to predict the need for transfusion or endoscopy; it has been found to be an effective tool in determining discharge for low-risk patients [5]. The AIMS65 score is based on five parameters: albumin, INR, mental status, systolic blood pressure, and age; it is a powerful score, particularly for predicting in-hospital mortality, complications, and costs [6]. It has been reported to perform better than the GBS and Rockall scores, particularly in predicting mortality [7].

In recent years, efforts have been made to develop different scores in addition to classic scoring systems. One such study is the Hemoglobin, Albumin, Lymphocyte, and Platelet (HALP) score, which was first defined by Chen et al. to predict gastric cancer prognosis [8]. HALP comprehensively reflects the patient’s hematological, inflammatory, and nutritional status. It has been shown to have prognostic value in oncological diseases as well as cardiovascular diseases, stroke, and critical illness [9,10,11,12,13]. The rationale for investigating the HALP score in UGIB is grounded in the pathophysiology of acute hemorrhage. Hemoglobin reflects blood loss severity, albumin indicates nutritional reserve, lymphocytes capture the immune response, and platelets reflect clotting capacity. Since all four parameters are routinely available in ED laboratories, this index offers a rapid, cost-free risk stratification tool. By simultaneously integrating these pathophysiological pathways, we hypothesized that the HALP score might offer a unique physiological perspective in predicting the stress response and recovery potential in acute UGIB, potentially overcoming some limitations of existing scores that focus primarily on hemodynamics or single laboratory parameters.

Although a recent UGIB study assessed HALP for 30-day mortality and rebleeding [14], its comparative performance for in-hospital mortality and intervention-related outcomes remains unclear. The main aim of this study was to evaluate and compare the prognostic performance of the HALP, GBS, AIMS65, and Rockall scores in predicting outcomes among patients with upper gastrointestinal bleeding.

## 2. Materials and Methods

### 2.1. Study Design

This study was designed as a single-center, retrospective observational study. The medical records of adult patients with endoscopy-confirmed UGIB who presented to the ED were retrospectively reviewed through the hospital information system. The data collection process included consecutive patients who presented to the ED between 1 January 2021, and 31 December 2024. This study was reported in accordance with the STROBE (Strengthening the Reporting of Observational Studies in Epidemiology) guidelines.

Ethics Approval and Consent to Participate

The study was conducted in accordance with the Declaration of Helsinki and approved by the Harran University Clinical Research Ethics Committee (approval number: HRÜ/25.16.33; date of approval: 29 September 2025). The requirement for individual informed consent was waived by the same committee because this was a retrospective study based on routinely collected clinical data.

### 2.2. Study Population and Selection Criteria

The study included patients aged 18 years and older with endoscopy-confirmed UGIB who initially presented to the ED during the study period. Eligibility criteria were as follows: (1) presentation to the ED during the study period with suspected UGIB, subsequently confirmed by endoscopy; and (2) availability of laboratory parameters required for calculating the HALP score, such as hemoglobin, albumin, lymphocyte, and platelet counts, at the time of presentation. The electronic medical records of the patients included in the study were reviewed in detail, and demographic and clinical data were obtained from the hospital automation system.

**Figure 1:**
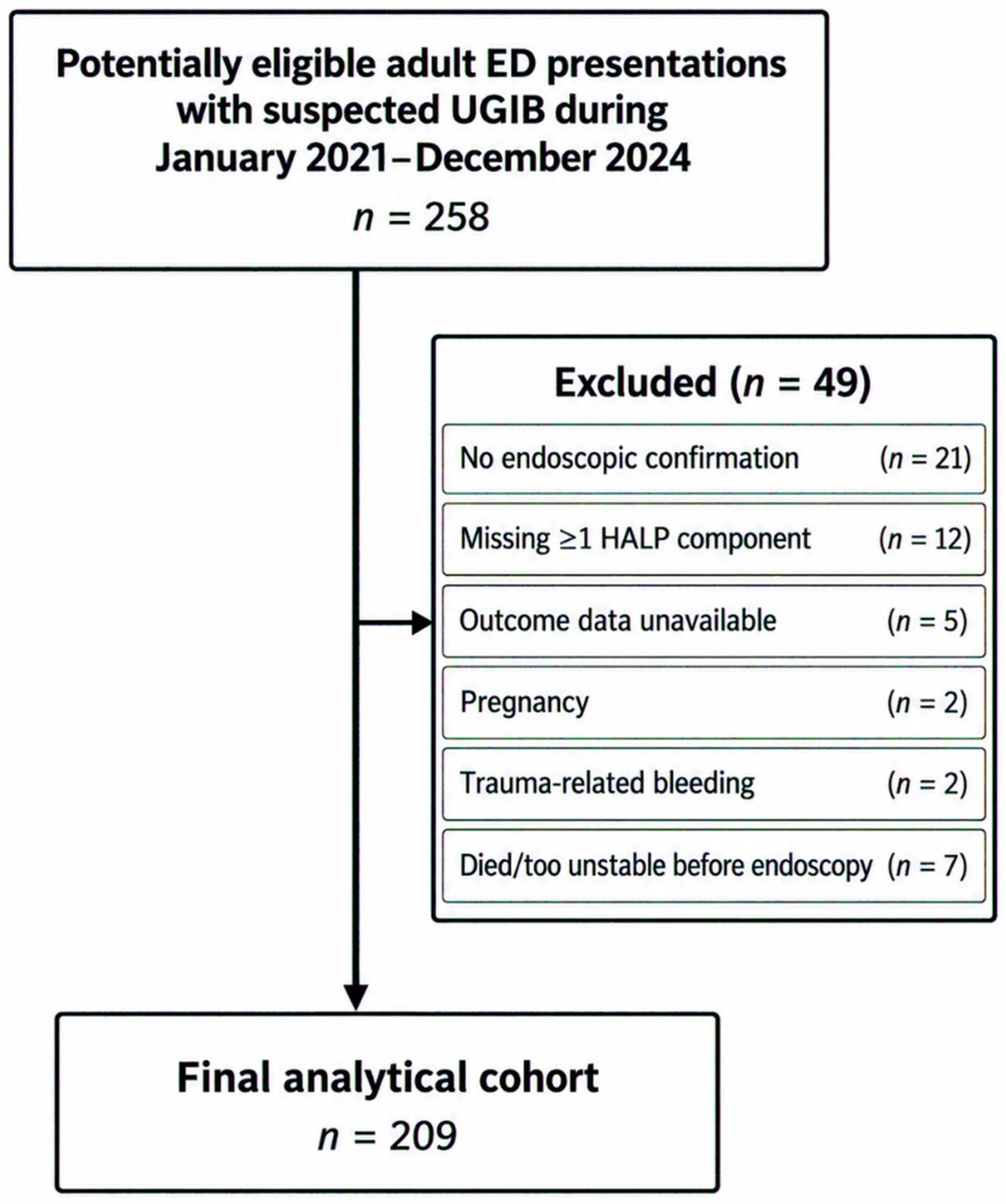
Flow diagram of patient screening, exclusions, and inclusion in the final analytical cohort (exclusion categories were mutually exclusive).

Exclusion criteria included pregnancy, trauma-related bleeding, unavailable study outcome data, and missing laboratory components required to calculate the HALP score. Because endoscopic confirmation was required, patients without confirmation were excluded; those who died before or were too unstable to undergo endoscopy were classified separately. Only the index presentation was included for patients with multiple eligible presentations; transferred patients were included only when initial-evaluation score variables and prespecified outcomes were reliably available. Figure 1 summarizes patient selection and mutually exclusive exclusion reasons.

### 2.3. Data Collection

From the recorded data, the patients’ age, sex, presenting complaint (hematemesis, melena, syncope, altered mental status), vital signs at the time of admission (blood pressure and pulse), comorbidities (heart failure, coronary artery disease, chronic kidney disease, chronic liver disease, diabetes mellitus, and malignancy) were recorded. Laboratory parameters included hemoglobin, hematocrit, platelet, leukocyte, lymphocyte, neutrophil, and monocyte counts; prothrombin time, activated partial thromboplastin time, and international normalized ratio (INR); urea, creatinine, aspartate aminotransferase, alanine aminotransferase, albumin, total bilirubin, sodium, potassium, C-reactive protein, lactate, and troponin levels. Laboratory values used for score calculation were obtained from the first blood samples collected before intravenous fluid administration or PRBC transfusion in all patients. This sequence was verified from the available records, including referring-hospital records for transferred patients.

Heart failure was defined based on documented clinical diagnosis in the patient’s medical records [15]. Chronic liver disease was defined as documented cirrhosis or chronic hepatitis in the medical records [16].

Endoscopic findings were obtained from the index endoscopy reports recorded in the hospital information system and classified as variceal or non-variceal bleeding. In addition, the number of packed red blood cell (PRBC) units transfused within the first 24 h, intensive care unit (ICU) admission, and in-hospital mortality were also recorded.

Definition of Clinical Outcomes:

In-hospital mortality was defined as death from any cause occurring during the index hospital admission. PRBC transfusion within 24 h was defined as the administration of at least one unit of PRBCs within the first 24 h following ED presentation. PRBC transfusions were guided by the treating team’s assessment of overall clinical status, not a study-specific protocol; retrospective records did not permit identification of a uniform hemoglobin threshold. A composite severe UGIB outcome was defined as either transfusion of ≥2 units of PRBCs within the first 24 h after ED presentation or UGIB-related ICU admission. UGIB-related ICU admission during the index hospitalization was determined jointly by the treating emergency medicine and intensive care physicians based on overall clinical assessment and the documented indication for intensive care. ICU admissions unrelated to the UGIB episode were not considered part of the composite endpoint. For the composite outcome, patients meeting either component were classified as having the outcome, and patients meeting both components were counted only once. All study scores were calculated retrospectively and were unavailable to treating clinicians; thus, they did not guide PRBC transfusion, ICU admission, or other management decisions.

### 2.4. Calculation of Scores

HALP Score

The HALP score was calculated as follows: hemoglobin (g/L) × serum albumin (g/L) × absolute lymphocyte count (×10^9^/L) ÷ platelet count (×10^9^/L) [8]. All four components were converted to these units before calculation, and the same unit convention was applied to all patients. This calculation yields HALP values on the conventional scale, without additional rescaling.

Other Risk Scores

- Glasgow–Blatchford Score: The GBS incorporates hemoglobin level, blood urea nitrogen (BUN), systolic blood pressure, pulse rate, history of melena or syncope, hepatic disease, and heart failure and is used to assess the risk of adverse outcomes in patients presenting to the ED. In our laboratory database, the urea parameter was reported as urea (mg/dL). Therefore, urea values were converted to BUN using the standard conversion factor (BUN [mg/dL] = urea [mg/dL] × 0.467), and the converted BUN values were used to calculate the GBS according to the original scoring criteria [5].
- AIMS65 Score: The AIMS65 score includes five criteria, each assigned one point: albumin < 30 g/L, international normalized ratio (INR) > 1.5, altered mental status, systolic blood pressure ≤ 90 mmHg, and age > 65 years. The total score ranges from 0 to 5 [6]. The AIMS65 was calculated from the initial assessment; altered mental status was defined as documented impairment of consciousness at emergency department presentation. For score calculation, syncope was assigned exclusively to the GBS, whereas altered mental status was assigned exclusively to AIMS65; these variables were not used interchangeably.
- Rockall Score: The full Rockall score was calculated according to the original scoring system using age, hemodynamic status (systolic blood pressure and heart rate), comorbidity, endoscopic diagnosis, and major stigmata of recent hemorrhage obtained from the index endoscopy report [4]. The full Rockall score incorporates endoscopic findings and was therefore calculated after index endoscopy, unlike HALP, GBS, and AIMS65, which are available at ED presentation. It was calculable for all patients (209/209 available and 0 missing for each component); missing findings were neither assumed absent nor assigned zero points.

### 2.5. Statistical Analysis

Statistical analyses were conducted using SPSS version 26.0 and RStudio 2026.04.0. Data visualization was performed using Python version 3.11. Continuous variables were summarized as mean ± standard deviation for normally distributed variables and as median with interquartile range (IQR) for non-normally distributed variables. The distribution of continuous variables was assessed using the Shapiro–Wilk test.

Comparisons of continuous variables were performed using the Mann–Whitney U test. Categorical variables were summarized as counts and percentages and compared using the Pearson chi-square test or Fisher’s exact test, as appropriate.

Among patients included in the final analytical cohort, variable-specific missingness was <3% for recorded study variables and was handled using available-case analysis. This estimate reflects missingness after cohort assembly only; it excludes patients omitted beforehand for missing eligibility-defining data, including HALP components.

Receiver operating characteristic (ROC) curves were constructed to evaluate the discriminatory performance of HALP, GBS, AIMS65, and Rockall for in-hospital mortality, PRBC transfusion within 24 h, and composite severe UGIB outcome. Area under the curve (AUC) values with 95% confidence intervals (CIs) were calculated. Optimal cutoff values were determined de novo using the Youden index, and sensitivity, specificity, PPV, and NPV at these cutoffs were reported with 95% confidence intervals calculated using the Wilson method. Because lower HALP values indicate higher risk, the ROC direction was reversed, whereas reported HALP cutoffs were expressed on the original score scale. The term “inverse HALP” refers only to this reversal of the ROC direction; no reciprocal (1/HALP) or other mathematical transformation was performed. Pairwise comparisons of AUCs were performed using the correlated-sample DeLong test. Each pairwise DeLong test was restricted to patients with complete data for both scores and the outcome, ensuring within-patient comparisons. These analyses were exploratory, and *p*-values were not adjusted for multiple comparisons.

Because this was a retrospective cohort study including all eligible patients during the predefined study period, no prospective sample size calculation was performed. Precision of the estimates was therefore assessed using 95% confidence intervals.

Univariable logistic regression analyses were first performed to identify candidate variables associated with in-hospital mortality. The multivariable analysis was intended to assess the adjusted association between HALP and in-hospital mortality rather than the incremental prognostic value of HALP beyond an established prediction model. A prespecified parsimonious multivariable model was constructed to minimize the risk of overfitting. The variable selection strategy combined clinical a priori reasoning with a statistical pre-screening step. Specifically, the following three rules were applied: (1) the HALP score was retained regardless of its statistical significance as the primary variable of interest; (2) age, systolic BP, and heart rate were included a priori based on their established prognostic importance in UGIB and their relevance to hemodynamic instability; and (3) additional candidate variables were considered for inclusion if they demonstrated an association with in-hospital mortality at a liberal threshold of *p* < 0.10 on univariable analysis and were considered clinically relevant. Albumin and INR were selected to represent nutritional/hepatic status and coagulation, respectively. Other variables associated with mortality in univariable analyses were not included because of the limited number of events and risk of overfitting. Because albumin is a component of HALP, the adjusted estimate is conditional on albumin and does not represent the independent effect of the composite score.

Firth’s penalized logistic regression was used for the multivariable analysis because of the limited number of in-hospital deaths and the resulting low events-per-variable ratio. This approach was selected to reduce small-sample bias and improve the stability of parameter estimates. However, Firth penalization does not eliminate the imprecision or limited statistical power associated with a small number of outcome events.

Multicollinearity was assessed using the variance inflation factor (VIF), with VIF values < 5 considered indicative of no substantial multicollinearity. Model fit was explored using the Hosmer–Lemeshow goodness-of-fit test; a nonsignificant result was not interpreted as evidence of satisfactory calibration.

All statistical tests were two-sided, and *p* < 0.05 was considered statistically significant.

## 3. Results

Among the 209 patients, 191 (91.4%) received at least one unit of PRBCs within the first 24 h. Eighteen patients (8.6%) received no PRBC transfusion, 29 (13.9%) received one unit, and 162 (77.5%) received ≥ 2 units. UGIB-related ICU admission occurred in 177 patients (84.7%), and 190 patients (90.9%) met the composite severe UGIB endpoint. In-hospital mortality occurred in 24 patients (11.5%), and variceal bleeding was identified in 36 patients (17.2%). Given the small variceal-bleeding subgroup, subgroup-specific prognostic analyses were not performed; overall findings should be interpreted in light of this case mix.

Age and sex distribution were comparable between groups (age, 63.0 [IQR, 42.8–76.0] vs. 66.5 [IQR, 58.0–78.0] years, *p* = 0.094; male sex, 73.5% vs. 62.5%, *p* = 0.373). In contrast, non-survivors had lower systolic blood pressure (86.5 [66.8–107.0] vs. 116.0 [104.0–125.0] mmHg, *p* < 0.001) and higher heart rates (124.5 [112.5–150.0] vs. 87.0 [75.0–95.0] beats/min, *p* < 0.001). Altered mental status or syncope was more frequent among non-survivors (50.0% vs. 10.8%, *p* < 0.001). Heart failure was also more common (37.5% vs. 18.9%), although the difference was not statistically significant (*p* = 0.067). Liver disease and melena prevalence were similar between groups (*p* = 0.995 and *p* = 0.689, respectively) (Table 1).

Laboratory values for hemoglobin, hematocrit, platelet and lymphocyte counts, ALT, total bilirubin, sodium, potassium, and troponin were comparable between groups (all *p* > 0.05). Conversely, non-survivors had higher leukocyte, neutrophil, monocyte, urea, creatinine, AST, CRP, and lactate levels, together with prolonged PT, aPTT, and INR, and lower albumin levels (*p* < 0.05 for all). Overall, non-survivors exhibited greater inflammation, renal dysfunction, coagulopathy, hyperlactatemia, and hypoalbuminemia (Table 2).

The Rockall, GBS, and AIMS65 scores were significantly higher in non-survivors than in survivors (7.0 [IQR, 5.75–8.00] vs. 5.0 [4.00–6.00]; 15.0 [12.0–17.0] vs. 12.0 [9.0–14.0]; and 2.0 [1.0–3.0] vs. 1.0 [0.0–1.0], respectively; all *p* < 0.001). Conversely, the HALP score was lower in non-survivors than in survivors (14.28 [9.37–32.40] vs. 20.43 [12.47–34.42]), although this difference was not statistically significant (*p* = 0.201) (Table 3).

**Figure 2:**
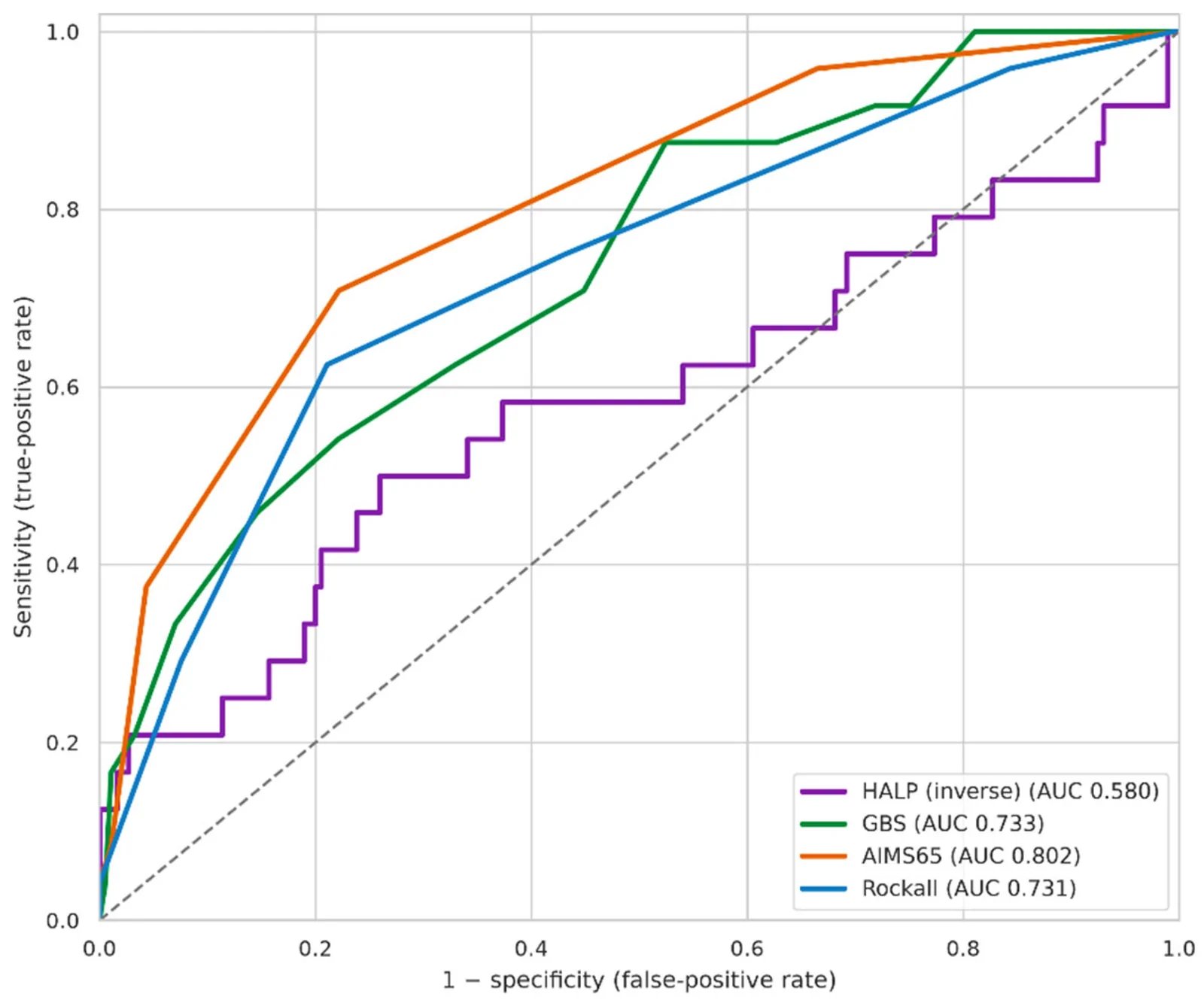
ROC curves for in-hospital mortality.

For in-hospital mortality, the AUCs were 0.802 (95% CI, 0.711–0.894) for AIMS65, 0.733 (0.626–0.840) for GBS, 0.731 (0.618–0.844) for Rockall, and 0.580 (0.436–0.725) for inverse HALP. At the Youden-derived cutoffs, sensitivity and specificity were 70.8% and 77.8% for AIMS65 ≥ 2, 87.5% and 47.6% for GBS ≥ 12, 62.5% and 78.9% for Rockall ≥7, and 50.0% and 74.1% for a HALP cutoff of ≤12.74 on the conventional scale. AIMS65 had a higher AUC than inverse HALP (*p* = 0.012), whereas GBS and Rockall did not differ significantly from inverse HALP (*p* = 0.116 and *p* = 0.142, respectively). AIMS65 did not differ from GBS or Rockall (*p* = 0.254 and *p* = 0.249), and GBS and Rockall were similar (*p* = 0.973) (Figure 2).

**Figure 3:**
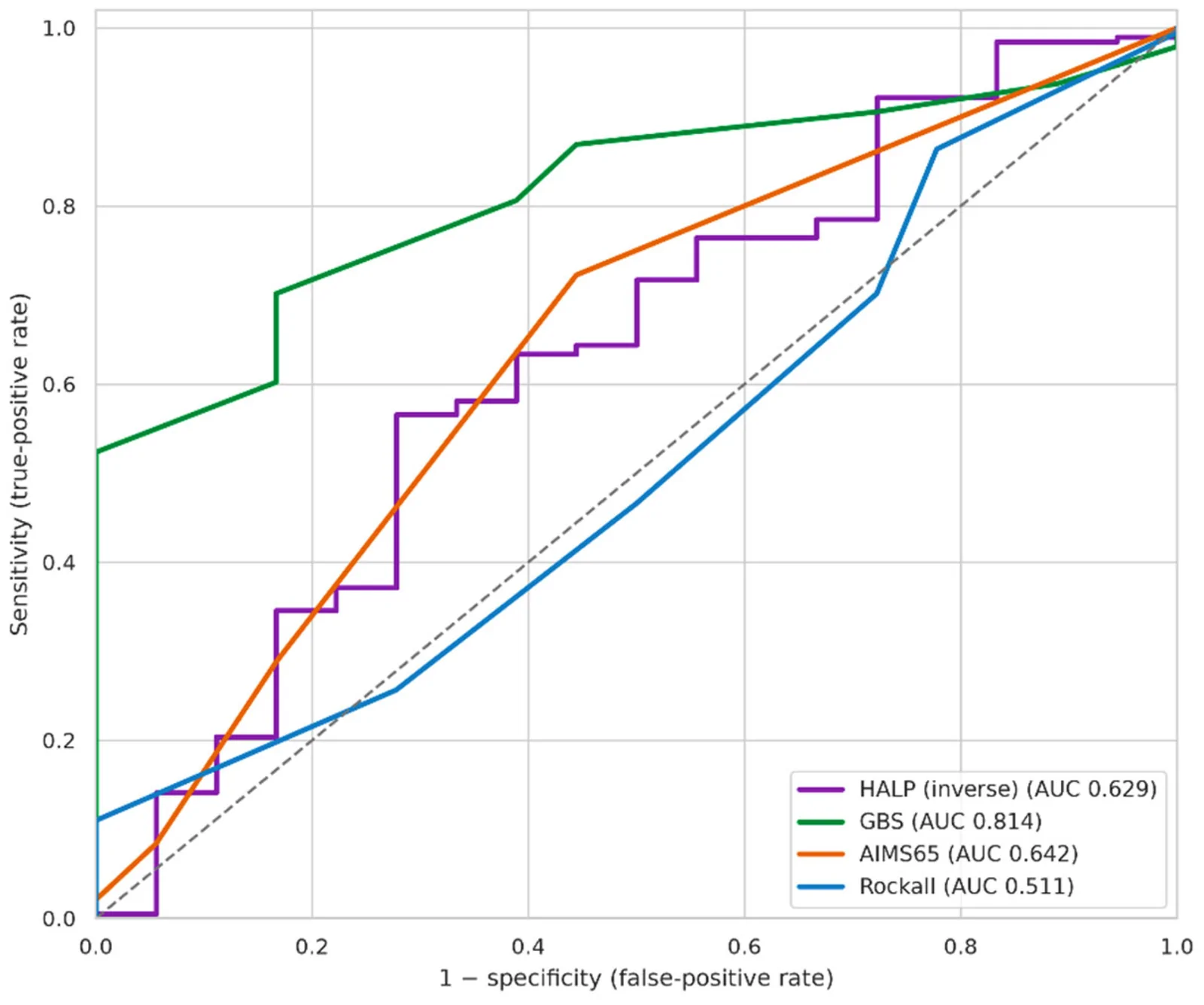
ROC curves for PRBC transfusion within 24 h.

For PRBC transfusion within 24 h, the AUCs were 0.814 (95% CI, 0.740–0.889) for GBS, 0.642 (0.508–0.777) for AIMS65, 0.629 (0.483–0.775) for inverse HALP, and 0.511 (0.371–0.651) for Rockall. The Youden-derived cutoffs were GBS ≥ 11, AIMS65 ≥ 1, HALP ≤ 21.40, and Rockall ≥ 8. GBS had a higher AUC than inverse HALP, AIMS65, and Rockall (*p* = 0.015, *p* = 0.009, and *p* < 0.001, respectively). AIMS65 and Rockall did not differ (*p* = 0.132), nor did inverse HALP differ from AIMS65 or Rockall (*p* = 0.890 and *p* = 0.162, respectively) (Figure 3).

**Figure 4:**
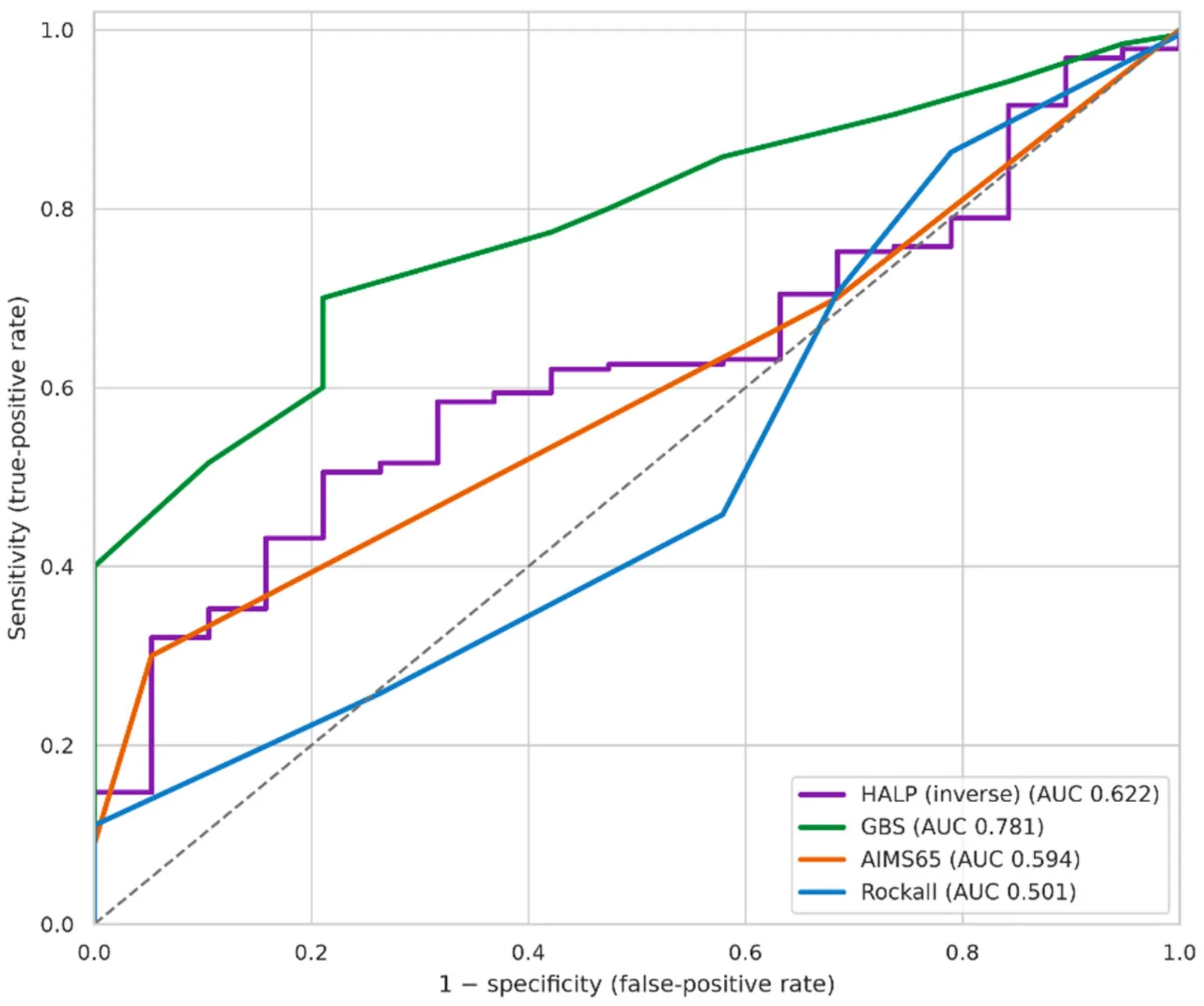
ROC curves for composite severe UGIB outcome.

For the composite severe UGIB outcome, the AUCs were 0.781 (95% CI, 0.695–0.866) for GBS, 0.622 (0.513–0.732) for inverse HALP, 0.594 (0.497–0.692) for AIMS65, and 0.501 (0.363–0.638) for Rockall. The selected cutoffs were GBS ≥ 11, HALP ≤ 19.14, AIMS65 ≥ 2, and Rockall ≥ 8. GBS had a higher AUC than inverse HALP, AIMS65, and Rockall (*p* = 0.006, *p* < 0.001, and *p* < 0.001, respectively), whereas inverse HALP did not differ from AIMS65 or Rockall (*p* = 0.656 and *p* = 0.138, respectively) (Figure 4).

All diagnostic performance estimates for the three prespecified clinical outcomes are provided in Table 4.

All pairwise DeLong comparisons for these outcomes are provided in Supplementary Table S1. The sensitivity analysis of the individual components of the composite severe UGIB outcome is presented in Supplementary Table S2. The corresponding 2 × 2 contingency tables for all four scores at the thresholds reported in Table 4 are provided in Supplementary Table S3.

Univariable logistic regression analyses identified candidate predictors of in-hospital mortality (Table 5). Systolic BP ≤ 90 mmHg (OR = 10.37), heart rate ≥ 100 beats/min (OR = 25.83), altered consciousness/syncope (OR = 8.25), heart failure (OR = 2.57), albumin < 30.0 g/L (OR = 4.62), and higher leukocyte (OR = 1.12 per 10^3^/µL), neutrophil (OR = 1.14 per 10^3^/µL), creatinine (OR = 1.68 per 1 mg/dL), lactate (OR = 1.19 per 1 mmol/L), and CRP levels (OR = 1.17 per 1 mg/dL) were significantly associated with in-hospital mortality (all *p* < 0.05). No statistically significant association between HALP and in-hospital mortality was detected (OR = 0.98, 95% CI 0.81–1.20, *p* = 0.864). INR > 1.5 (*p* = 0.059) met the pre-specified *p* < 0.10 criterion and was included in the multivariable model. Although age > 65 years did not meet either criterion (*p* = 0.300), it was included because of its established clinical relevance as an in-hospital mortality predictor in UGIB. Among the established prognostic scores, GBS (OR = 1.31), AIMS65 (OR = 3.28), and Rockall (OR = 1.71) were significantly associated with in-hospital mortality (all *p* < 0.001), whereas no statistically significant association was detected for HALP.

Firth-penalized multivariable logistic regression showed that systolic BP ≤ 90 mmHg (OR = 8.83, 95% CI 2.63–29.62, *p* < 0.001) and heart rate ≥ 100 beats/min (OR = 24.62, 95% CI 6.73–90.09, *p* < 0.001) remained independently associated with in-hospital mortality. Albumin < 30.0 g/L was not significant after adjustment (OR = 3.16, 95% CI 0.91–10.96, *p* = 0.070), nor were age > 65 years (OR = 1.70, *p* = 0.417) or INR > 1.5 (OR = 2.86, *p* = 0.124). The association between HALP score and in-hospital mortality was not statistically significant (aOR per 10-point increase = 0.91, 95% CI: 0.66–1.26, *p* = 0.571). The Hosmer–Lemeshow goodness-of-fit test yielded χ^2^ = 14.28 (df = 8, *p* = 0.075). The complete model is presented in Table 6.

## 4. Discussion

In this single-center retrospective cohort of 209 adults with endoscopy-confirmed UGIB, inverse HALP showed limited discrimination for in-hospital mortality, PRBC transfusion within 24 h, and composite severe UGIB (AUCs, 0.580, 0.629, and 0.622, respectively). AIMS65 had the highest mortality AUC (0.802), but did not significantly outperform GBS or Rockall. The full Rockall score showed moderate discrimination for mortality (AUC, 0.731) but poor discrimination for the other outcomes. GBS had the highest discrimination for PRBC transfusion and composite severe UGIB, significantly outperforming inverse HALP, AIMS65, and Rockall for both outcomes. Overall, score performance was outcome-specific rather than uniformly hierarchical.

The observed in-hospital mortality was 11.5%, comparable to rates reported in contemporary UGIB cohorts, although direct comparisons are limited by differences in case mix, endoscopic confirmation, variceal bleeding prevalence, referral patterns, and outcome definitions [17]. Non-survivors had lower systolic blood pressure, higher heart rate, and more frequent altered consciousness or syncope than survivors, consistent with impaired circulatory reserve and early shock physiology. Systolic blood pressure and heart rate remained independently associated with in-hospital mortality in the Firth-penalized model. These findings support using risk scores to complement—not replace—immediate bedside assessment and resuscitation in hemodynamically unstable patients.

Non-survivors also showed a laboratory profile consistent with greater systemic physiological disturbance, including higher leukocyte, neutrophil, and monocyte counts; prolonged PT/aPTT and higher INR; increased urea, creatinine, AST, CRP, and lactate; and lower albumin. These variables are not interchangeable markers of bleeding severity and may reflect hypoperfusion, acute inflammation, organ dysfunction, liver disease, comorbidity, or treatment-related changes. Although albumin was substantially lower among non-survivors and showed a borderline association after adjustment, it was not an independent predictor of in-hospital mortality in the final model, supporting its role as a clinically relevant risk marker rather than an independent prognostic factor.

AIMS65 had the highest mortality AUC (0.802), with a Youden-derived cutoff of ≥2. However, its AUC did not differ significantly from GBS (*p* = 0.254) or Rockall (*p* = 0.249). This finding is consistent with the score’s derivation and validation as a mortality-prediction tool based on admission variables. Its five components—albumin, INR, altered mental status, systolic blood pressure, and age—capture several of the physiological domains that were prominent in this cohort [6]. External studies have reported heterogeneous rankings according to population characteristics, the proportion of variceal bleeding, and whether mortality or intervention was evaluated. For example, a prospective comparison found an association between AIMS65 and mortality, whereas GBS was not independently associated with mortality in the overall cohort; performance for other outcomes varied across scores [18].

GBS performed best for PRBC transfusion within 24 h and the composite severe UGIB outcome. This is clinically plausible because GBS incorporates hemoglobin, urea, pulse, systolic blood pressure, melena, syncope, hepatic disease, and heart failure—variables reflecting both the physiological consequences of bleeding and the likelihood of hospital-based treatment [5]. Its original purpose was to predict treatment requirements, and subsequent evidence has supported its use for intervention-related outcomes and for identifying very-low-risk patients who may not require hospital-based management [5,17]. A 2025 systematic review and meta-analysis likewise found higher pooled discrimination with GBS than with Rockall for transfusion and surgical intervention, whereas the scores did not differ materially for mortality or rebleeding [19]. However, the present study did not assess outpatient discharge safety, and the post hoc cutoff of ≥11 should not be interpreted as a universal treatment threshold.

The composite severe UGIB outcome was defined as either transfusion of ≥2 units of PRBCs within the first 24 h after ED presentation or UGIB-related ICU admission. It is therefore an intervention-based composite that reflects treatment intensity and institutional triage decisions in addition to biological hemorrhage severity. Composite definitions vary across UGIB risk-score studies, and guidelines have emphasized this lack of uniformity [20]. The superior performance of GBS may partly reflect the conceptual alignment between its components and intervention-related outcomes. Conversely, the lower performance of AIMS65 and Rockall should not be interpreted as evidence that these scores lack clinical value; rather, their components were not designed to predict this specific combination of transfusion and ICU admission. Because PRBC transfusion within 24 h was defined as receipt of at least one unit, interpretations should clearly distinguish this outcome from the ≥2-unit transfusion component of the composite severe UGIB outcome. The composite outcome was highly prevalent because UGIB-related ICU admission and transfusion of ≥2 PRBC units were common and often co-occurred. Thus, the high PPV and low NPV should be interpreted in light of this prevalence, not as prevalence-independent measures of score performance. The small number of patients without the outcome also limits the precision of specificity and NPV estimates.

The full Rockall score showed moderate discrimination for in-hospital mortality but poor discrimination for PRBC transfusion within 24 h and the composite severe UGIB outcome. This pattern should be interpreted in light of score timing: unlike GBS and AIMS65, the full Rockall score incorporates endoscopic diagnosis and stigmata and is therefore a post-endoscopic score rather than a purely pre-endoscopic emergency-department tool [4]. Its principal value may emerge after the bleeding source and endoscopic characteristics have been established. The present findings do not invalidate its original prognostic role; rather, they argue against describing the full Rockall score as an initial ED triage instrument. Comparisons between pre-endoscopic and post-endoscopic scores should consequently be framed as comparisons of different clinical information sets, not as tests of universally interchangeable tools.

The limited discrimination of HALP across all three prespecified outcomes contrasts with its rationale as an immunonutritional and inflammatory index. HALP was originally developed in patients with gastric carcinoma, in whom hemoglobin, albumin, lymphocyte count, and platelet count were associated with long-term survival [8]. Acute UGIB differs physiologically from that setting because outcomes are rapidly influenced by circulatory compromise, bleeding rate and source, coagulopathy, endoscopic stigmata, resuscitation, transfusion, and treatment response. A multiplicative score based on four laboratory components may therefore capture baseline or systemic physiological status without adequately representing the time-sensitive determinants of acute hemorrhage. This is a plausible mechanistic explanation for the weak discrimination observed here, but it does not establish a causal pathway.

Recent UGIB-specific evidence should also be considered. In a 2025 retrospective study of 256 patients with confirmed UGIB, HALP had an AUC of 0.772 for 30-day mortality, comparable to Rockall, and an AUC of 0.688 for rebleeding versus 0.739 for Rockall; the authors proposed HALP as a complement to established scores [14]. The difference from the present findings is not necessarily contradictory because the studies differed in mortality horizon, outcome definitions, sample size, comparator scores, case mix, and treatment pathways. Here, inverse HALP had an AUC of 0.580 for in-hospital mortality and was evaluated for PRBC transfusion within 24 h and the composite severe UGIB outcome rather than rebleeding. These heterogeneous results suggest context-dependent prognostic behavior but do not support routine use of HALP as a stand-alone tool for acute UGIB without external validation.

DeLong comparisons showed that GBS significantly outperformed inverse HALP, AIMS65, and Rockall for PRBC transfusion within 24 h and composite severe UGIB. For in-hospital mortality, AIMS65 significantly outperformed inverse HALP, while no other pairwise differences were statistically significant. Inverse HALP’s AUC was not the lowest for every outcome, and nonsignificant pairwise comparisons do not establish equivalence or non-inferiority. Because pairwise comparisons were not adjusted for multiplicity, these findings should be interpreted cautiously as exploratory.

**Strengths and Limitations**

This study compared HALP with three established UGIB scores in the same clinically characterized cohort, limiting differences in populations and outcome definitions. It assessed in-hospital mortality, 24-h PRBC transfusion, and a clinically relevant composite severe-UGIB outcome, covering multiple aspects of acute care. Endoscopic confirmation strengthened diagnostic certainty and enabled calculation of the complete Rockall score. ROC analyses with correlated-sample DeLong tests formally compared discrimination, while the prespecified parsimonious Firth-penalized model reduced small-sample bias versus conventional maximum-likelihood regression.

Several limitations should be acknowledged. The single-center retrospective design limits generalizability and remains vulnerable to selection bias, information bias, incomplete documentation, and variation in the timing of laboratory and vital-sign measurements. Missing data accounted for less than 3% of recorded variables and were handled using available-case analysis, but residual bias related to missingness cannot be excluded. Only in-hospital mortality, PRBC transfusion within 24 h, and the composite severe UGIB outcome were assessed; therefore, performance for 30-day or 6-month mortality, rebleeding, and readmission remains uncertain. ICU admission, which contributed to the composite severe UGIB outcome, was based on treating physicians’ judgment and may reflect institutional policies, resource availability, and center-specific classification practices rather than biological severity alone.

Although UGIB was endoscopically confirmed and the endoscopic data required to calculate the full Rockall score were available, detailed data on bleeding etiology, hemostatic interventions, endoscopy timing, antithrombotic therapy, cirrhosis severity, and adjudicated causes of death were inconsistent, precluding detailed etiologic or treatment subgroup analyses. This is particularly relevant to HALP, as cirrhosis and variceal bleeding may independently affect albumin and platelet levels. The results cannot be generalized to lower gastrointestinal bleeding, and inconsistent symptom documentation permitted reliable quantitative analysis only of melena. Although score-calculation samples were obtained before intravenous fluids or PRBC transfusion in all patients, subsequent resuscitation and transfusion were not standardized or fully quantified and may have affected outcomes. Because hemoglobin contributes to GBS and informs transfusion decisions, GBS performance for transfusion may partly reflect treatment-decision incorporation. Incomplete information on endoscopy timing, pharmacological management, treatment intensity, and changes in practice over the study period also leaves the possibility of residual treatment-related confounding.

Endoscopic confirmation may have introduced survival and selection bias by excluding patients who died before endoscopy or were too unstable to undergo the procedure. Accordingly, findings apply only to patients who survived and were stable enough for endoscopic confirmation and may underestimate risk in the sickest patients with suspected UGIB. The multivariable in-hospital mortality model should also be interpreted cautiously because there were only 24 in-hospital mortality events, corresponding to approximately four events per predictor included in the final model. Firth penalization reduces finite-sample bias but cannot overcome the limitations of low event numbers, reduced power for subgroup analyses, or wide confidence intervals. Finally, the ROC cutoffs were derived post hoc using the Youden index and evaluated in the same cohort, which may have led to optimistic estimates of performance at the selected thresholds. Bootstrap-based internal validation was not performed, and the stability of these cutoffs under resampling was not assessed. These cutoffs should therefore be considered exploratory and require validation in independent cohorts before clinical application.

## 5. Conclusions

In this endoscopy-confirmed UGIB cohort, inverse HALP showed limited discrimination across the prespecified outcomes and no statistically significant adjusted association with in-hospital mortality. AIMS65 had the highest mortality AUC, without significant superiority over GBS or Rockall, whereas GBS performed best for transfusion and composite severe UGIB; the full Rockall score showed moderate discrimination for mortality but poor discrimination for the intervention-related outcomes.

These findings support selecting risk scores according to the intended clinical outcome rather than assuming universal superiority of a single instrument. Although HALP may have context-dependent prognostic value, the present findings do not support its use as a stand-alone risk-stratification tool in acute UGIB. Given conflicting evidence from UGIB-specific studies and the limitations of this single-center cohort, these results should be considered hypothesis-generating. Prospective multicenter studies with standardized data collection and treatment protocols, prespecified endpoints, an adequate number of in-hospital mortality events, and external validation are needed before HALP or any cohort-derived cutoff can be incorporated into a broadly applicable clinical decision rule.

## Figures and Tables

**Table 1:** Demographic and clinical characteristics according to in-hospital mortality.

Variable	Survivors (*n* = 185)	Non-Survivors (*n* = 24)	*p*-Value
Age (years) (IQR)	63.00 (42.75–76.00)	66.50 (58.00–78.00)	0.094
Sex (Male) *n* (%)	136 (73.5%)	15 (62.5%)	0.373
Systolic BP (mmHg) (IQR)	116.00 (104.00–125.00)	86.50 (66.75–107.00)	<0.001
Heart rate (beats/min) (IQR)	87.00 (75.00–95.00)	124.50 (112.50–150.00)	<0.001
Altered consciousness/syncope *n* (%)	20 (10.8%)	12 (50.0%)	<0.001
Heart failure *n* (%)	35 (18.9%)	9 (37.5%)	0.067
Liver disease *n* (%)	43 (23.2%)	5 (20.8%)	0.995
Melena *n* (%)	127 (68.6%)	18 (75.0%)	0.689

Survivors: discharged alive; non-survivors: died during the index hospitalization; BP: Blood Pressure; IQR: Interquartile range; Categorical variables compared by Pearson chi-square or Fisher’s exact test; continuous variables by Mann–Whitney U test. The combined altered consciousness/syncope variable was used only for descriptive comparisons and univariable regression analysis.

**Table 2:** Laboratory findings according to in-hospital mortality.

Variable	Survivors (*n* = 185)	Non-Survivors (*n* = 24)	*p*-Value
Hemoglobin (g/L)	86.0 (71.0–100.0)	75.0 (65.0–114.3)	0.799
Hematocrit (%)	26.00 (21.80–31.00)	23.40 (21.00–35.25)	1.000
Platelet (×10^3^/µL)	222.00 (165.00–272.00)	189.50 (131.75–307.50)	0.590
Leukocyte (×10^3^/µL)	10.00 (7.90–12.00)	13.00 (9.97–18.20)	0.004
Lymphocyte (×10^3^/µL)	1.60 (1.10–2.28)	1.10 (0.89–2.05)	0.201
Neutrophil (×10^3^/µL)	7.20 (5.60–9.50)	10.75 (6.17–16.00)	0.014
Monocyte (×10^3^/µL)	0.58 (0.40–0.76)	0.71 (0.53–1.10)	0.019
PT (s)	13.30 (12.20–14.80)	15.00 (13.15–18.95)	0.003
aPTT (s)	22.50 (20.30–25.30)	25.40 (23.00–30.25)	0.014
INR	1.14 (1.02–1.30)	1.36 (1.14–1.67)	0.009
Urea (mg/dL)	67.15 (42.00–107.00)	102.00 (61.25–175.00)	0.007
Creatinine (mg/dL)	0.83 (0.69–1.17)	1.46 (0.93–3.10)	<0.001
AST (U/L)	19.00 (13.00–29.00)	50.50 (19.75–83.25)	<0.001
ALT (U/L)	15.00 (10.00–23.00)	19.50 (9.75–38.50)	0.172
Albumin (g/L)	34.0 (30.0–38.0)	28.5 (25.8–31.5)	<0.001
Total bilirubin (mg/dL)	0.67 (0.40–0.98)	0.67 (0.37–1.90)	0.764
Sodium (mmol/L)	137.00 (135.00–140.00)	137.50 (134.00–141.25)	0.901
Potassium (mmol/L)	4.30 (4.00–4.66)	4.59 (4.15–5.20)	0.067
CRP (mg/dL)	0.73 (0.20–2.65)	4.38 (0.57–9.75)	0.001
Lactate (mmol/L)	1.72 (1.46–2.14)	2.92 (1.98–4.90)	<0.001
Troponin (ng/L)	12.00 (4.00–34.00)	40.00 (0.00–157.50)	0.142

Values expressed as median (IQR). PT: Prothrombin time; aPTT: Activated partial thromboplastin time; INR: International Normalized Ratio; AST: Aspartate aminotransferase; ALT: Alanine aminotransferase; CRP: C-reactive protein. All comparisons by Mann–Whitney U test.

**Table 3:** Prognostic score distributions according to in-hospital mortality.

Score	Survivors (*n* = 185)	Non-Survivors (*n* = 24)	*p*-Value
HALP score	20.43 (12.47–34.42)	14.28 (9.37–32.40)	0.201
Rockall score	5.00 (4.00–6.00)	7.00 (5.75–8.00)	<0.001
GBS score	12.00 (9.00–14.00)	15.00 (12.00–17.00)	<0.001
AIMS65 score	1.00 (0.00–1.00)	2.00 (1.00–3.00)	<0.001

Values expressed as median (IQR). HALP: Hemoglobin, Albumin, Lymphocytes, Platelet score; GBS: Glasgow–Blatchford Score; AIMS65: Age > 65, INR > 1.5, Mental status, Systolic BP ≤ 90 mmHg, Albumin < 30.0 g/L. All comparisons by Mann–Whitney U test.

**Table 4:** ROC performance of the four scores for the three prespecified clinical outcomes.

Outcome	Score	AUC (95% CI)	Optimal Cutoff	Sensitivity, % (95% CI)	Specificity, % (95% CI)	PPV, % (95% CI)	NPV, % (95% CI)
In-hospital mortality	HALP, inverse	0.580 (0.436–0.725)	≤12.74	50.0 (31.4–68.6)	74.1 (67.3–79.8)	20.0 (11.8–31.8)	91.9 (86.5–95.3)
In-hospital mortality	GBS	0.733 (0.626–0.840)	≥12	87.5 (69.0–95.7)	47.6 (40.5–54.7)	17.8 (11.9–25.7)	96.7 (90.8–98.9)
In-hospital mortality	AIMS65	0.802 (0.711–0.894)	≥2	70.8 (50.8–85.1)	77.8 (71.3–83.2)	29.3 (19.2–42.0)	95.4 (90.7–97.7)
In-hospital mortality	Rockall	0.731 (0.618–0.844)	≥7	62.5 (42.7–78.8)	78.9 (72.5–84.2)	27.8 (17.6–40.9)	94.2 (89.3–96.9)
PRBC transfusion within 24 h	HALP, inverse	0.629 (0.483–0.775)	≤21.40	56.5 (49.5–63.4)	72.2 (49.1–87.5)	95.6 (90.1–98.1)	13.5 (8.1–21.8)
PRBC transfusion within 24 h	GBS	0.814 (0.740–0.889)	≥11	70.2 (63.3–76.2)	83.3 (60.8–94.2)	97.8 (93.8–99.3)	20.8 (13.1–31.6)
PRBC transfusion within 24 h	AIMS65	0.642 (0.508–0.777)	≥1	72.3 (65.5–78.1)	55.6 (33.7–75.4)	94.5 (89.6–97.2)	15.9 (8.9–26.8)
PRBC transfusion within 24 h	Rockall	0.511 (0.371–0.651)	≥8	11.0 (7.3–16.2)	100.0 (82.4–100.0)	100.0 (84.5–100.0)	9.6 (6.1–14.6)
Composite severe UGIB outcome	HALP, inverse	0.622 (0.513–0.732)	≤19.14	50.5 (43.5–57.6)	78.9 (56.7–91.5)	96.0 (90.2–98.4)	13.8 (8.5–21.5)
Composite severe UGIB outcome	GBS	0.781 (0.695–0.866)	≥11	70.0 (63.1–76.1)	78.9 (56.7–91.5)	97.1 (92.7–98.9)	20.8 (13.1–31.6)
Composite severe UGIB outcome	AIMS65	0.594 (0.497–0.692)	≥2	30.0 (23.9–36.9)	94.7 (75.4–99.1)	98.3 (90.9–99.7)	11.9 (7.7–18.1)
Composite severe UGIB outcome	Rockall	0.501 (0.363–0.638)	≥8	11.1 (7.3–16.3)	100.0 (83.2–100.0)	100.0 (84.5–100.0)	10.1 (6.6–15.2)

CI, confidence interval; AUC, area under the receiver operating characteristic curve; PPV, positive predictive value; NPV, negative predictive value. HALP was analyzed in the inverse direction because lower HALP values indicate higher risk. Cutoffs are reported on the original score scale. Sensitivity, specificity, PPV, and NPV are presented as percentages with 95% confidence intervals calculated using the Wilson method. All analyses in Table 4 included 209 patients. Event/non-event counts were 24/185 for in-hospital mortality, 191/18 for receipt of ≥1 unit of PRBCs within 24 h, and 190/19 for the composite severe UGIB outcome.

**Table 5:** Univariable Logistic Regression Analysis for Predictors of In-Hospital Mortality.

Variable	OR	95% CI	*p*-Value
Age > 65 years	1.58	0.67–3.73	0.300
Systolic BP ≤ 90 mmHg	10.37	4.11–26.17	<0.001
Heart rate ≥ 100 beats/min	25.83	8.24–80.98	<0.001
Altered consciousness/syncope	8.25	3.27–20.81	<0.001
Heart failure	2.57	1.04–6.35	0.041
Albumin < 30.0 g/L	4.62	1.92–11.15	<0.001
INR > 1.5	2.56	0.96–6.80	0.059
Leukocyte (per 10^3^/µL)	1.12	1.05–1.21	0.001
Neutrophil (per 10^3^/µL)	1.14	1.05–1.23	0.001
Creatinine (per 1 mg/dL)	1.68	1.24–2.28	<0.001
Lactate (per 1 mmol/L)	1.19	1.03–1.37	0.015
CRP (per 1 mg/dL)	1.17	1.08–1.27	<0.001
HALP (per 10-point increase)	0.98	0.81–1.20	0.864
GBS (per 1 point)	1.31	1.13–1.52	<0.001
AIMS65 (per 1 point)	3.28	2.03–5.30	<0.001
Rockall (per 1 point)	1.71	1.29–2.26	<0.001

OR, odds ratio. HALP was analyzed on the conventional scale and expressed per 10-point increase.

**Table 6:** Firth-penalized multivariable logistic regression analysis for in-hospital mortality.

Variable	OR	95% CI	*p*-Value
Age > 65 years	1.70	0.47–6.15	0.417
Systolic BP ≤ 90 mmHg	8.83	2.63–29.62	<0.001
Heart rate ≥ 100 beats/min	24.62	6.73–90.09	<0.001
Albumin < 30.0 g/L	3.16	0.91–10.96	0.070
INR > 1.5	2.86	0.75–10.94	0.124
HALP (per 10-point increase)	0.91	0.66–1.26	0.571

VIF, variance inflation factor. Hosmer–Lemeshow goodness-of-fit test: χ^2^ = 14.28, df = 8, *p* = 0.075. VIF values for all variables were <5. Firth-penalized logistic regression was used to reduce small-sample bias given the limited number of mortality events (EPV = 4.0). HALP was modeled in its conventional direction; an OR < 1 indicates lower odds of in-hospital mortality with increasing HALP.

## Data Availability

The data presented in this study are available on request from the corresponding author due to patient privacy and confidentiality considerations.

## Supplementary Materials

The following supporting information can be downloaded at: https://www.mdpi.com/article/10.3390/jcm15208196/s1, Table S1: Pairwise DeLong comparisons of AUCs for the three prespecified clinical outcomes; Table S2: ROC performance of the four scores for the individual components and the composite severe UGIB outcome; Table S3: Two by two contingency counts for the score thresholds reported in Table 4.

## Abbreviations

AIMS65, albumin, international normalized ratio, altered mental status, systolic blood pressure, and age > 65 years; ALT, alanine aminotransferase; aPTT, activated partial thromboplastin time; AST, aspartate aminotransferase; AUC, area under the receiver operating characteristic curve; BP, blood pressure; BUN, blood urea nitrogen; CI, confidence interval; CRP, C-reactive protein; ED, emergency department; GBS, Glasgow–Blatchford Score; HALP, hemoglobin–albumin–lymphocyte–platelet score; ICU, intensive care unit; INR, international normalized ratio; IQR, interquartile range; NPV, negative predictive value; OR, odds ratio; PPV, positive predictive value; PRBCs, packed red blood cells; PT, prothrombin time; ROC, receiver operating characteristic; SBP, systolic blood pressure; UGIB, upper gastrointestinal bleeding; VIF, variance inflation factor.
