# Supplementary material for: Outcome-Specific Performance of HALP, AIMS65, Glasgow–Blatchford, and Rockall Scores in Emergency Department Patients with Acute Upper Gastrointestinal Bleeding: A Retrospective Cohort Study
Source: J Clin Med. 2026 Oct 21;15(20):8196. doi: 10.3390/jcm15208196 (PMC13705710; doi:10.3390/jcm15208196)
Supplement: Supplementary file 1 [file jcm-15-08196-s001.zip › jcm-4611144-supplementary.pdf]

Supplementary Table S1: Pairwise DeLong comparisons of AUCs for the three prespecified clinical outcomes

**Outcome**	**Score 1**	**Score 2**	**AUC 1**	**AUC 2**	**AUC difference**	**p-value**
In-hospital mortality	HALP, inverse	GBS	0.580	0.733	−0.153	0.116
	HALP, inverse	AIMS65	0.580	0.802	−0.222	0.012
	HALP, inverse	Rockall	0.580	0.731	−0.151	0.142
	GBS	AIMS65	0.733	0.802	−0.069	0.254
	GBS	Rockall	0.733	0.731	0.002	0.973
	AIMS65	Rockall	0.802	0.731	0.071	0.249
PRBC transfusion within 24 h	HALP, inverse	GBS	0.629	0.814	−0.185	0.015
	HALP, inverse	AIMS65	0.629	0.642	−0.013	0.890
	HALP, inverse	Rockall	0.629	0.511	0.118	0.162
	GBS	AIMS65	0.814	0.642	0.172	0.009
	GBS	Rockall	0.814	0.511	0.304	<0.001
	AIMS65	Rockall	0.642	0.511	0.131	0.132
Composite severe UGIB outcome	HALP, inverse	GBS	0.622	0.781	−0.158	0.006
	HALP, inverse	AIMS65	0.622	0.594	0.028	0.656
	HALP, inverse	Rockall	0.622	0.501	0.122	0.138
	GBS	AIMS65	0.781	0.594	0.186	<0.001
	GBS	Rockall	0.781	0.501	0.280	<0.001
	AIMS65	Rockall	0.594	0.501	0.094	0.188

AUC, area under the receiver operating characteristic curve; GBS, Glasgow–Blatchford Score; HALP, hemoglobin–albumin–lymphocyte–platelet score; PRBC, packed red blood cells; UGIB, upper gastrointestinal bleeding. HALP was analyzed in the inverse direction because lower HALP values indicate higher risk. AUC differences were calculated as AUC 1 minus AUC 2. Event/non-event counts were 24/185 for in-hospital mortality, 191/18 for receipt of ≥1 unit of PRBCs within 24 hours, and 190/19 for the composite severe UGIB outcome. Pairwise comparisons were performed using DeLong's test. Pairwise comparisons were exploratory, and p-values were not adjusted for multiple comparisons. A two-sided p-value < 0.05 was considered statistically significant.

Supplementary Table S2: ROC performance of the four scores for the individual components and the composite severe UGIB outcome

Outcome	Score	Events (n)	AUC (95% CI)
≥2 PRBC units within 24 h	HALP, inverse	162	0.605 (0.513–0.696)
	GBS	162	0.658 (0.570–0.745)
	AIMS65	162	0.529 (0.446–0.611)
	Rockall	162	0.475 (0.386–0.564)
UGIB-related ICU admission	HALP, inverse	177	0.617 (0.526–0.707)
	GBS	177	0.764 (0.685–0.843)
	AIMS65	177	0.648 (0.569–0.726)
	Rockall	177	0.471 (0.363–0.579)
Composite severe UGIB outcome	HALP, inverse	190	0.622 (0.513–0.732)
	GBS	190	0.781 (0.695–0.866)
	AIMS65	190	0.594 (0.497–0.692)
	Rockall	190	0.501 (0.363–0.638)

AUC, area under the receiver operating characteristic curve; CI, confidence interval; GBS, Glasgow–Blatchford Score; HALP, hemoglobin–albumin–lymphocyte–platelet score; ICU, intensive care unit; PRBC, packed red blood cells; UGIB, upper gastrointestinal bleeding. HALP was analyzed in the inverse direction because lower HALP values indicate higher risk. The composite severe UGIB outcome was defined as transfusion of ≥2 PRBC units within 24 h and/or UGIB-related ICU admission.

Supplementary Table S3: Two by two contingency counts for the score thresholds reported in Table 4

**Outcome**	**Score**	**Cutoff**	**TP**	**FP**	**FN**	**TN**
In-hospital mortality	HALP, inverse	≤12.74	12	48	12	137
In-hospital mortality	GBS	≥12	21	97	3	88
In-hospital mortality	AIMS65	≥2	17	41	7	144
In-hospital mortality	Rockall	≥7	15	39	9	146
PRBC transfusion within 24 h	HALP, inverse	≤21.40	108	5	83	13
PRBC transfusion within 24 h	GBS	≥11	134	3	57	15
PRBC transfusion within 24 h	AIMS65	≥1	138	8	53	10
PRBC transfusion within 24 h	Rockall	≥8	21	0	170	18
Composite severe UGIB outcome	HALP, inverse	≤19.14	96	4	94	15
Composite severe UGIB outcome	GBS	≥11	133	4	57	15
Composite severe UGIB outcome	AIMS65	≥2	57	1	133	18
Composite severe UGIB outcome	Rockall	≥8	21	0	169	19

TP, true positive; FP, false positive; FN, false negative; TN, true negative; GBS, Glasgow–Blatchford Score; HALP, hemoglobin–albumin–lymphocyte–platelet score; ICU, intensive care unit; PRBC, packed red blood cells; UGIB, upper gastrointestinal bleeding. All values are patient counts. Each row reports the four cells of a 2 × 2 contingency table, with 209 patients included in each analysis. Event/non-event counts were 24/185 for in-hospital mortality, 191/18 for receipt of ≥1 PRBC unit within 24 hours, and 190/19 for the composite endpoint. A positive classification satisfies the listed cutoff. HALP cutoffs are expressed on the original scale, with lower values indicating higher risk; higher values indicate higher risk for the other scores. The composite endpoint comprised transfusion of ≥2 PRBC units within 24 hours and/or UGIB-related ICU admission during the index hospitalization.
